# Understanding the interplay of colorectal cancer awareness and attitudes among Palestinians: a national cross-sectional study

**DOI:** 10.1186/s12885-024-12357-9

**Published:** 2024-05-15

**Authors:** Mohamedraed Elshami, Mohammad Fuad Dwikat, Ibrahim Al-Slaibi, Mohammed Alser, Maram Elena Albandak, Mohammed Ayyad, Shoruq Ahmed Naji, Balqees Mustafa Mohamad, Wejdan Sudki Isleem, Adela Shurrab, Bashar Yaghi, Yahya Ayyash Qabaja, Fatma Khader Hamdan, Raneen Raed Sweity, Remah Tayseer Jneed, Khayria Ali Assaf, Mohammed Madhat Hmaid, Iyas Imad Awwad, Belal Khalil Alhabil, Marah Naser Alarda, Amani Saleh Alsattari, Moumen Sameer Aboyousef, Omar Abdallah Aljbour, Rinad AlSharif, Christy Teddy Giacaman, Ali Younis Alnaga, Ranin Mufid Abu Nemer, Nada Mahmoud Almadhoun, Sondos Mahmoud Skaik, Shurouq I. Albarqi, Nasser Abu-El-Noor, Bettina Bottcher

**Affiliations:** 1 Division of Surgical Oncology, Department of Surgery, University Hospitals Cleveland Medical Center, 11100 Euclid Avenue, Lakeside, Cleveland, OH 7100, 44106 USA; 2 Ministry of Health, Gaza, Palestine; 3 Department of Internships, An-Najah National University Hospital, Nablus, Palestine; 4 Almakassed Hospital, Jerusalem, Palestine; 5 The United Nations Relief and Works Agency for Palestine Refugees in the Near East (UNRWA), Gaza, Palestine; 6 Faculty of Medicine, Al-Quds University, Jerusalem, Palestine; 7 Faculty of Pharmacy, Al-Azhar University of Gaza, Gaza, Palestine; 8 Doctors Without Borders (Médecins Sans Frontières), Hebron, Palestine; 9 Faculty of Medicine, Islamic University of Gaza, Gaza, Palestine; 10 Nasser Medical Complex, Khanyounis, Palestine; 11 Faculty of Medicine, An-Najah National University, Nablus, Palestine; 12 Faculty of Dentistry, Arab American University, Jenin, Palestine; 13 Augusta Victoria Hospital, Jerusalem, Palestine; 14 Faculty of Allied Medical Sciences, Arab American University, Jenin, Palestine; 15 Faculty of Medicine, Al-Azhar University, Gaza, Palestine; 16 Faculty of Nursing, Islamic University of Gaza, Gaza, Palestine

**Keywords:** Colorectal cancer, Attitudes, Health education, Symptoms, Risk factors, Early presentation, Palestine

## Abstract

**Background:**

In Palestine, colorectal cancer (CRC) is the second most common cause of cancer-related mortality after lung cancer. No studies have examined the relationship between CRC awareness and attitudes. This study aimed to investigate the interplay between CRC awareness and attitudes among the Palestinian population.

**Methods:**

A nationwide cross-sectional survey was carried out between July 2019 and March 2020. Convenience sampling was used to collect data from hospitals, primary healthcare facilities, and public areas in 11 governorates. Modified, translated-into-Arabic versions of the validated Bowel Cancer Awareness Measure and Cancer Awareness Measure-Mythical Causes Scale were utilized to assess the awareness of CRC signs/symptoms, risk factors, and causation myths. The cumulative awareness score for each domain was computed and stratified into tertiles. The top tertile denoted ‘high’ awareness, while the remaining two tertiles denoted ‘low’ awareness.

**Results:**

The final analysis included 4,623 participants; of whom, 3115 (67.4%) reported positive attitudes toward CRC. In total, 1,849 participants (40.0%) had high awareness of CRC signs/symptoms. There was no association between displaying a high awareness of CRC signs/symptoms and having positive attitudes toward CRC. A total of 1,840 participants (38.9%) showed high awareness of CRC risk factors. Participants with high CRC risk factor awareness were more likely to display positive attitudes toward CRC (OR = 1.22, 95% CI: 1.07–1.39). Only 219 participants (4.7%) had high awareness of CRC causation myths. Participants with high awareness of CRC causation myths were more likely to exhibit positive attitudes toward CRC (OR = 2.48, 95% CI: 1.71–3.58).

**Conclusion:**

A high awareness level of CRC risk factors and causation myths was associated with a greater likelihood of demonstrating positive attitudes toward CRC in terms of perceived susceptibility, importance of early detection, and consequences of developing the disease. Future educational interventions should focus on raising public awareness about CRC, with a particular emphasis on risk factors and causation myths, to maximize the potential for shaping favorable attitudes toward the disease.

## Background

Colorectal cancer (CRC) is the second most prevalent cancer with 1.45 million cases diagnosed in 2020 [1]. In Palestine, CRC is the second most common cancer constituting 12.9% of cancer cases. Furthermore, CRC is the second most common cause of cancer-related mortality accounting for 14.3% of cancer-related deaths [2]. The growing incidence of CRC worldwide can also be seen in the Middle East. According to data from the International Agency for Research on Cancer, the Eastern Mediterranean Region is predicted to have the second-highest rise in fatalities related to CRC between 2020 and 2040 [3] .

There are several risk factors for CRC that can be classified into modifiable and non-modifiable factors. Modifiable risk factors include lack of physical activity, obesity, low-fiber diet, high consumption of red meat, smoking, and excessive alcohol consumption [4]. Non-modifiable factors include older age, family history of CRC, and type 2 diabetes mellitus [4]. Signs/symptoms that could be suggestive of CRC include blood per rectum, changes in bowel habits, iron deficiency anemia, abdominal pain, and the presence of abdominal or rectal masses [5].

A person’s attitude is a reflection of how they see and assess a particular matter or course of action, taking into account its consequences for themselves [6]. The attitude toward a certain disease is crucial to the adoption and maintenance of certain patterns of health behavior. It indicates a propensity for self-care behaviors that can help in reducing stress related to the disease, increase acceptance of treatment, improve the self-esteem of patients, as well as allow a more positive perception of health [7]. In addition, previous studies have indicated that personal beliefs and attitudes are accurate indicators of a patient’s capacity to manage that illness [8].

It has also been shown that the perception of disease severity and appraisal of symptoms stand out as major factors affecting delays in diagnosis [9]. For example, asymptomatic presentation and lack of acute intolerable pain can often hinder presentation to primary care facilities [10, 11]. Other barriers to help-seeking for possible CRC symptoms include the willingness to use herbs, fear of disease, distrust in medical professionals, lack of social support, and poor health literacy [12–14].

Negative attitudes toward CRC often include beliefs that it is incurable or that available treatment might be worse than the actual disease, while positive attitudes include beliefs in effective treatment and better quality of life thereafter as well as beliefs in effective screening for CRC [15, 16]. Therefore, positive attitudes may positively influence help-seeking behavior, increase participation in screening as well as lead to adoption of lifestyle choices known to reduce CRC risk factors [17–19]. This may eventually help reduce the incidence of CRC and the associated mortality [20]. It was also demonstrated that individuals having positive attitudes toward cancer were more likely to receive educational information about cancer to increase their awareness [21]. Other studies found that negative attitudes and beliefs toward cancer limited the possibility of cancer control [22, 23].

The awareness of CRC signs/symptoms, risk factors, and causation myths was found to be low in Palestine [24–26]. However, to our knowledge, no study in Palestine has investigated the relationship between CRC awareness and attitudes. Therefore, this study aimed to examine the interplay between CRC awareness and attitudes toward this disease.

## Materials and methods

### Study design and population

This cross-sectional study was carried out from July 2019 to March 2020. Palestine is divided into 16 governorates, including five in the Gaza Strip and 11 in the West Bank and Jerusalem. In 2019, there were around 2.6 million adults, accounting for 51.6% of the entire Palestinian population (almost 5 million) [27]. Thus, responses were collected from adult (≥ 18 years) Palestinians living in the West Bank and Jerusalem or the Gaza Strip. Public areas, primary healthcare centers, and government hospitals served as recruitment sites to collect responses from participants.

### Sampling methods

Convenience sampling was employed to recruit participants from governmental hospitals, primary healthcare centers, and public areas in the corresponding 11 governorates (four from the Gaza Strip and seven from the West Bank and Jerusalem) out of 16 governorates in Palestine. Public spaces included parks, shopping centers, places of worship, transportation stations, downtowns, and others. The goal was to increase the representativeness of the study cohort by recruiting participants from different governorates and locations [24–26]. In 2019, the Palestinian population aged 15 and above was estimated at 3,109,063 individuals [28]. With a confidence level of 95.0%, an absolute error of 2.0%, and a type I error rate of 5.0%, a minimum sample size of 2401 participants was required to detect a 50% overall positive attitude toward CRC.

### Inclusion and exclusion criteria

To be eligible to participate in the study, participants had to be Palestinian adults (≥ 18 years old) visiting one of the designated sites for data collection. Exclusion criteria included possessing a citizenship other than Palestinian, being a visitor to oncology departments or clinics, being employed or a student in a health-related field, and inability to complete the questionnaire.

### Data collection and measurement tool

In this study, two validated assessment tools were utilized for data collection. Namely, the Bowel Cancer Awareness Measure (BoCAM) [29], and the Cancer Awareness Measure-Mythical Causes Scale (CAM-MYCS) [30], were modified and translated to gather data on public awareness of CRC signs/symptoms, risk factors, and causation myths after translation to Arabic. Cancer Research in the UK and University College London produced the first version of both BoCAM and CAM-MYCS. Both questionnaires are verified instruments for determining public knowledge about CRC [29, 30]. The questionnaires were translated from English into Arabic and then back-translated into English by two different bilingual healthcare professionals in each stage. All those healthcare professionals had relevant expertise in survey design and clinical research. The accuracy of translation and content validity of the questionnaire were assessed by five independent experts in the fields of gastroenterology, coloproctology, and public health. Subsequently, a pilot study with 25 participants was carried out to ensure the clarity of the questions in the Arabic questionnaire. The final analysis excluded the data gathered in the pilot study. Cronbach’s alpha was used to evaluate the internal consistency of the questionnaire, and a value of 0.80 indicated that it was satisfactory.

There were five sections in the questionnaire. The first section covered sociodemographic factors including age, gender, educational attainment, employment status, monthly income, marital status, place of residence, presence of chronic health condition, knowledge of someone with cancer, and site of data collection. The second section evaluated participants’ awareness regarding 12 signs/symptoms associated with CRC. Respondents were prompted to respond on a 5-point Likert scale, ranging from 1 (strongly disagree) to 5 (strongly agree). In the third section, participants were enquired about their awareness of 11 CRC risk factors, employing the same aforementioned 5-point Likert scale. Of the 12 signs/symptoms related to CRC, nine were retained from the original BoCAM [29] and three additional symptoms, namely ‘feeling persistently full,’ ‘unexplained loss of appetite,’ and ‘unexplained generalized fatigue,’ were incorporated based on other versions of the Cancer Awareness Measure [31, 32]. Similarly, 10 risk factors were adapted from the original BoCAM, with an extra risk factor specifically pertaining to ‘smoking cigarettes’ included due to its prevalence in the Palestinian population [33]. The fourth section assessed the participants’ ability in identifying 13 myths of CRC causation x as incorrect. Among those 13 myths, 12 were adapted from the original CAM-MYCS [30], and an additional myth concerning ‘eating burnt food’ was included due to its prevalence as a belief within the Palestinian community. The fifth section included 11 questions about attitudes toward CRC, adapted from previous studies [34–37], and employing the same aforementioned 5-point Likert scale.

All participants were invited for a face-to-face interview to complete the questionnaire. Data were collected using Kobo Toolbox, a safe and user-friendly smartphone application [38]. Prior to data collection, data collectors were trained on the utilization of the Kobo Toolbox, approaching study participants, and facilitating the completion of the questionnaire.

### Statistical analysis

Participant characteristics were summarized using descriptive statistics. For non-normally distributed continuous variables, the median and interquartile range (IQR) were employed, while frequencies and percentages were utilized to describe categorical variables.

Prompt recognition of CRC signs/symptoms and risk factors was assessed using a 5-point Likert scale (1 = strongly disagree, 5 = strongly agree). Correct responses were categorized as ‘strongly agree’ or ‘agree,’ while ‘strongly disagree,’ ‘disagree,’ or ‘not sure’ were deemed incorrect. Additionally, respondents were queried about myths related to the causation of CRC, and answers expressing disagreement (‘disagree’ or ‘strongly disagree’) were considered correct, with all other responses considered incorrect.

The assessment of participants’ awareness regarding CRC signs/symptoms, risk factors, and causation myths employed a scoring system that had also been utilized in previous studies [24–26]. Participants were given one point for each correctly identified item. Subsequently, the cumulative awareness score for each domain was computed and stratified into tertiles. The top tertile denoted ‘high’ awareness, while the remaining two tertiles were designated as ‘low’ awareness. Similarly, participants were given one point for answering with ‘agree’ or ‘strongly agree’ on each of the questions related to attitudes toward CRC. The total attitude score was calculated. The median attitude score was utilized to dichotomize the continuous overall attitude score; a score ≤ 4 was considered ‘negative’ attitude and a score ≥ 5 was considered ‘positive’ attitude.

Pearson’s Chi-square test was performed to examine the association between demonstrating high awareness in each domain and agreeing with questions pertaining to positive attitudes toward CRC. Subsequently, a multivariable logistic regression was conducted to account for various covariates, including age, gender, education level, employment status, monthly income, marital status, place of residence, presence of a chronic disease, knowing someone with cancer, and site of data collection. This model was determined a priori based on previous studies [24–26]. Similar analyses were also performed to examine the association between demonstrating high awareness in each domain and showing a positive attitude toward CRC.

Missing data were hypothesized to be missed completely at random and thus, complete case analysis was utilized to handle them. Data were analyzed using Stata software version 17.0 (StataCorp, College Station, Texas, United States).

## Results

### Participant characteristics

Out of 5,254 individuals invited to participate, 4,877 agreed and completed the questionnaire (response rate = 92.3%). A total of 254 questionnaires were excluded; 44 did not match the inclusion criteria and 210 had missing data. Therefore, the final analysis included 4,623 questionnaires. Participants had a median age (IQR) of 31.0 years [24.0–43.0] and 1,879 (40.6%) of them were men **(**Table 1**)**.

**Table 1:** Characteristics of study participants

Characteristic	Total (*n* = 4623)
Age, median [IQR]	31.0 [24.0, 43.0]
Age group, n (%)	
18 to 44	3608 (78.1)
45 or older	1015 (21.9)
Male gender, n (%)	1879 (40.6)
Educational level, n (%)	
Secondary or below	2217 (47.9)
Post–secondary	2406 (52.1)
Occupation, n (%)	
Unemployed/housewife	2067 (44.7)
Employed	1898 (41.1)
Retired	96 (2.1)
Student	562 (12.1)
Monthly income ≥ 1450 NIS, n (%)	3039 (65.7)
Marital status, n (%)	
Single	1414 (30.5)
Married	3067 (66.4)
Divorced/Widowed	142 (3.1)
Residency, n (%) Gaza Strip West Bank and Jerusalem	1923 (41.6) 2700 (58.4)
Having a chronic disease, n (%)	906 (19.6)
Knowing someone with cancer, n (%)	2395 (51.8)
Site of data collection, n (%)	
Public Spaces	1450 (31.4)
Hospitals	1659 (35.9)
Primary healthcare centers	1514 (33.7)

n = number of participants, IQR = interquartile range.

### CRC symptom awareness and attitudes toward CRC

In total, 1849 study participants (40.0%) demonstrated high CRC symptom awareness (Table 2). Participants with high CRC symptom awareness were more likely than those with low awareness to agree on four out of 11 questions related to CRC, namely ‘early detection of CRC increases the possibility of more effective treatment’ (OR = 1.79, 95% CI: 1.45–2.22), ‘early detection of CRC increases the chances of survival’ (OR = 1.49, 95% CI: 1.23–1.79), ‘CRC is not an infectious disease’ (OR = 1.36, 95% CI: 1.16–1.59), and ‘would live longer than 5 years if diagnosed with CRC’ (OR = 1.39, 95% CI: 1.22–1.58).

**Table 2:** Summary of association between demonstrating high awareness of colorectal cancer signs and symptoms and the attitudes toward colorectal cancer among study participants

Question	Low awareness (*N* = 2774) *n* (%)	High awareness (*N* = 1849) *n* (%)	OR* (95% CI)	*p*–value
Early detection of colorectal cancer increases the possibility of more effective treatment.	2426 (87.5)	1720 (93.0)	1.79 (1.45–2.22)	< 0.001
Early detection of colorectal cancer increases the chances of survival.	2360 (85.1)	1654 (89.5)	1.49 (1.23–1.79)	< 0.001
Colorectal cancer is not an infectious disease.	2190 (78.9)	1565 (84.6)	1.36 (1.16 1.59)	< 0.001
Taking herbs is not a cure for colorectal cancer.	1380 (49.7)	884 (47.8)	0.89 (0.79-1.00)	0.054
Colorectal cancer would not threaten your relationship with your (future) spouse.	1037 (37.4)	722 (39.0)	1.01 (0.89–1.14)	0.93
The problems that you would experience with colorectal cancer would not last for a long time.	1043 (37.6)	710 (38.4)	1.00 (0.88–1.13)	0.99
Your chances of getting colorectal cancer in the next few years are not high.	1135 (40.9)	678 (36.7)	0.87 (0.77–0.98)	0.026
The thought of colorectal cancer does not scare you.	924 (33.3)	669 (36.2)	1.06 (0.93–1.20)	0.38
If you developed colorectal cancer, you would not feel that the therapy makes you sicker than the disease itself.	776 (28.0)	513 (27.7)	0.98 (0.86–1.12)	0.82
You will not get colorectal cancer sometime during your life.	936 (33.7)	535 (28.9)	0.82 (0.72–0.93)	0.003
If you developed colorectal cancer, you would live longer than 5 years.	772 (27.8)	670 (36.2)	1.39 (1.22–1.58)	< 0.001

n = number of participants, OR = odds ratio, CI = confidence interval.

*Adjusted for age, gender, education level, employment status, monthly income, marital status, place of residence, presence of a chronic disease, knowing someone with cancer, and site of data collection.

‘Early detection of CRC increases the possibility of more effective treatment’ was the most reported attitude to be agreed on by participants with low (*n* = 2426, 87.5%) or high (*n* = 1720, 93.0%) CRC symptom awareness. On the other hand, ‘would live longer than 5 years if diagnosed with CRC’ was the least reported attitude to be agreed on by participants with low CRC symptom awareness (*n* = 772, 27.8%), whereas it was ‘not feeling that therapy makes a participant sicker than the disease itself if they develop it’ in those with high awareness (*n* = 513, 27.7%).

### CRC risk factor awareness and attitudes toward CRC

A total of 1840 participants (39.8%) demonstrated high CRC risk factor awareness (Table 3). Participants with high CRC risk factor awareness were more likely than those with low awareness to agree on four out of 11 questions related to CRC, namely ‘early detection of CRC increases the possibility of more effective treatment’ (OR = 1.84, 95% CI: 1.49–2.28), ‘early detection of CRC increases the chances of survival’ (OR = 1.43, 95% CI: 1.19–1.71), ‘CRC is not an infectious disease’ (OR = 1.25, 95% CI: 1.07–1.46), and ‘would live longer than 5 years if diagnosed with CRC’ (OR = 1.33, 95% CI: 1.16–1.51).

**Table 3:** Summary of association between demonstrating high awareness of colorectal cancer risk factors and the attitudes toward colorectal cancer among study participants

Question	Low awareness (*N* = 2783) *n* (%)	High awareness (*N* = 1840) *n* (%)	OR* (95% CI)	*p*–value
Early detection of colorectal cancer increases the possibility of more effective treatment.	2439 (87.6)	1707 (92.8)	1.84 (1.49–2.28)	< 0.001
Early detection of colorectal cancer increases the chances of survival.	2377 (85.4)	1637 (89.0)	1.43 (1.19–1.71)	< 0.001
Colorectal cancer is not an infectious disease.	2223 (79.9)	1532 (83.3)	1.25 (1.07–1.46)	0.005
Taking herbs is not a cure for colorectal cancer.	1329 (47.8)	935 (50.8)	1.12 (0.99–1.26)	0.07
Colorectal cancer would not threaten your relationship with your (future) spouse.	1073 (38.6)	686 (37.3)	0.94 (0.83–1.06)	0.47
The problems that you would experience with colorectal cancer would not last for a long time.	1070 (38.4)	683 (37.1)	0.98 (0.86–1.11)	0.74
Your chances of getting colorectal cancer in the next few years are not high.	1075 (38.6)	738 (40.1)	1.05 (0.93–1.19)	0.42
The thought of colorectal cancer does not scare you.	990 (35.6)	603 (32.8)	0.94 (0.83–1.07)	0.37
If you developed colorectal cancer, you would not feel that the therapy makes you sicker than the disease itself.	790 (28.4)	499 (27.1)	0.95 (0.83–1.09)	0.47
You will not get colorectal cancer sometime during your life.	879 (31.6)	592 (32.2)	1.02 (0.90–1.16)	0.83
If you developed colorectal cancer, you would live longer than 5 years.	783 (28.1)	659 (35.8)	1.33 (1.16–1.51)	< 0.001

n = number of participants, OR = odds ratio, CI = confidence interval.

*Adjusted for age, gender, education level, employment status, monthly income, marital status, place of residence, presence of a chronic disease, knowing someone with cancer, and site of data collection.

Most participants with low (*n* = 2439, 87.6%) or high (*n* = 1707, 92.8%) CRC risk factor awareness agreed that ‘early detection of CRC increases the possibility of more effective treatment’. On the contrary, ‘would live longer than 5 years if diagnosed with CRC’ was the least reported attitude to be agreed on by participants with low CRC risk factor awareness (*n* = 783, 28.1%), whereas it was ‘not feeling that therapy makes a participant sicker than the disease itself if they develop it’ in those with high awareness (*n* = 499, 27.1%).

### CRC causation myth awareness and attitudes toward CRC

Only 219 participants (4.7%) displayed high awareness of CRC causation myths (Table 4). Participants with high awareness of CRC causation myths were more likely than those with low awareness to agree on seven out of 11 questions related to CRC.

**Table 4:** Summary of association between demonstrating high awareness of colorectal cancer causation myths and the attitudes toward colorectal cancer among study participants

Question	Low awareness (*N* = 4404) *n* (%)	High awareness (*N* = 219) *n* (%)	OR* (95% CI)	*p*–value
Early detection of colorectal cancer increases the possibility of more effective treatment.	3956 (89.8)	190 (86.8)	0.73 (0.48–1.10)	0.13
Early detection of colorectal cancer increases the chances of survival.	3831 (87.0)	183 (83.6)	0.77 (0.53–1.12)	0.17
Colorectal cancer is not an infectious disease.	3573 (81.1)	182 (83.1)	1.18 (0.82–1.71)	0.37
Taking herbs is not a cure for colorectal cancer.	2118 (48.1)	146 (66.7)	2.17 (1.62–2.90)	< 0.001
Colorectal cancer would not threaten your relationship with your (future) spouse.	1658 (37.6)	101 (46.1)	1.38 (1.04–1.82)	0.025
The problems that you would experience with colorectal cancer would not last for a long time.	1643 (37.3)	110 (50.2)	1.63 (1.24–2.15)	0.001
Your chances of getting colorectal cancer in the next few years are not high.	1689 (38.4)	124 (56.6)	2.33 (1.76–3.09)	< 0.001
The thought of colorectal cancer does not scare you.	1490 (33.8)	103 (47.0)	1.43 (1.08–1.89)	0.013
If you developed colorectal cancer, you would not feel that the therapy makes you sicker than the disease itself.	1205 (27.4)	84 (38.4)	1.63 (1.23–2.17)	0.001
You will not get colorectal cancer sometime during your life.	1358 (30.8)	113 (51.6)	2.42 (1.83–3.20)	< 0.001
If you developed colorectal cancer, you would live longer than 5 years.	1372 (31.2)	70 (32.0)	1.01 (0.75–1.36)	0.96

n = number of participants, OR = odds ratio, CI = confidence interval.

*Adjusted for age, gender, education level, employment status, monthly income, marital status, place of residence, presence of a chronic disease, knowing someone with cancer, and site of data collection.

The majority of participants with low (*n* = 3956, 89.8%) or high (*n* = 190, 86.8%) awareness of CRC causation myths agreed that ‘early detection of CRC increases the possibility of more effective treatment’. Conversely, the least commonly endorsed attitude in participants with low awareness of CRC causation myths was the belief that ‘not feeling that therapy makes a participant sicker than the disease itself if they develop it’ (*n* = 1205, 27.4%), whereas it was ‘would live longer than 5 years if diagnosed with CRC’ (*n* = 70, 32.0%) in participants with high awareness.

### Association between High CRC awareness and positive attitudes toward CRC

Overall, 3115 participants (67.4%) reported positive attitudes toward CRC. Participants with a high level of CRC risk factor awareness were more likely than those with low awareness to display a positive attitude toward CRC (70.1% vs. 65.6%, OR = 1.22, 95% CI: 1.07–1.39) **(**Table 5). Likewise, participants with high awareness of CRC causation myths were more likely than those with low awareness to show a positive attitude toward CRC (83.6% vs. 66.6%, OR = 2.48, 95% CI: 1.71–3.58). There was no association between demonstrating a high awareness of CRC signs and symptoms and showing a positive attitude toward CRC.

**Table 5:** Association of demonstrating high awareness in each domain with showing positive attitude toward colorectal cancer

Attitudes toward CRC	Low CRC symptom awareness (*N* = 2774) *n* (%)	High CRC symptom awareness (*N* = 1849) *n* (%)	OR* (95% CI)	*p*–value
Negative Positive	973 (34.1) 1827 (65.9)	561 (30.3) 1288 (69.7)	1.12 (0.98–1.27)	0.09

**Attitudes toward CRC**	**Low CRC risk factor awareness** **(** ***N*** ** = 2783)** n (%)	**High CRC risk factor awareness** **(** ***N*** ** = 1840)** n (%)	OR* (95% CI)	**p–value**
Negative Positive	957 (34.4) 1826 (65.6)	551 (29.9) 1289 (70.1)	1.22 (1.07–1.39)	0.003

**Attitudes toward CRC**	**Low CRC causation myth awareness** **(** ***N*** ** = 4404)** n (%)	**High CRC causation myth awareness** **(** ***N*** ** = 219)** n (%)	OR* (95% CI)	**p–value**
Negative Positive	1472 (33.4) 2932 (66.6)	36 (16.4) 183 (83.6)	2.48 (1.71–3.58)	< 0.001

n = number of participants, OR = odds ratio, CI = confidence interval, CRC = colorectal cancer.

*Adjusted for age, gender, education level, employment status, monthly income, marital status, place of residence, presence of a chronic disease, knowing someone with cancer, and site of data collection.

## Discussion

In this study, participants with a high level of awareness of CRC risk factors or causation myths were more likely than those with lower awareness to show a positive attitude toward CRC. In contrast, there was no association between having a high level of awareness of CRC signs/symptoms and exhibiting a positive attitude toward CRC. These findings emphasize the importance of increasing the awareness of particular areas regarding CRC to encourage people to have a positive attitude, which may influence their perception of the disease and their lifestyle choices.

There is no established CRC screening program in Palestine [2]. In all three domains of CRC awareness, most participants with high awareness agreed that ‘early detection of CRC increases the chances of survival’. This is similar to a study from Saudi Arabia that found more than 90% of participants believed that high survival rates are linked to early detection of CRC by colonoscopy [39]. This indicates that the general public believes in the efficacy of early detection. Such a positive attitude could be considered a good indicator of the acceptability of colonoscopy as a screening tool when establishing a nationwide screening program in Palestine.

Only one-third of participants with high awareness believed that they ‘would live longer than 5 years if diagnosed with CRC’. This could be related to the belief of fatalism, which is a common thought in non-Western countries where the person believes he/she has no control over their health and attributes their health events to religion or luck [40]. Cancer-fatalism is the belief that death is inevitable when cancer is present. Cancer screening, detection, and therapy are hampered by cancer fatalism [41, 42]. In Palestine, fatalism has been associated with a decreased tendency to undergo colonoscopy [43]. Similarly, a previous study from Lebanon found that 28% of participants refused to undergo CRC screening due to fatalism [44]. The majority of people in Palestine are religious [45]. Therefore, future campaigns discussing CRC screening and early detection should consider including the religious aspect to encourage people to undergo colonoscopy and to talk about their symptoms when they experience them. This is especially important given that the Islamic and Christian faiths, which are the major religions in Palestine, favor the use of medication and early detection and advocate for health-seeking [46–48].

A prior study from Oman, an Arabic country that shares similar cultural and religious background with Palestine, revealed that a lack of awareness regarding cancer signs/symptoms led to delays in seeking medical advice. This delay seemed to result from individuals’ difficulty interpreting their complaints as potential signs/symptoms of cancer, which influenced their attitude toward seeking healthcare [49]. In this study, there was no association between having a good awareness of CRC signs/symptoms and showing a positive attitude toward CRC. This coupled with the poor knowledge of CRC signs/symptoms indicates a problem in public education and is a warning sign that may lead to delayed presentation and late diagnosis [50]. It also highlights the importance of establishing a nationwide screening program for CRC in Palestine as people seem unable to promptly recognize CRC signs/symptoms when they occur. Policymakers in Palestine can learn from a previous experience in the United Arab Emirates. Al-Sharbatti and colleagues found that almost 85% of participants in the United Arab Emirates had little to no understanding of CRC warning signs and symptoms [51]. However, the Emirati government implemented a nationwide CRC screening program that has been running for many years and has been showing direct benefits to patients and community [52].

In this study, participants with a higher level of awareness of CRC risk factors and causation myths were more likely to show a positive attitude toward the disease. This is critical as such a positive attitude toward CRC may encourage people to modify their lifestyle to potentially lower their chances of having the disease [53]. Several risk factors of CRC can be modified when people are more aware of their attribution to developing CRC. For example, a previous study from the United States showed that over 50% of current smokers who heard or saw information related to the significance of quitting smoking and its role in lowering the risk of CRC seriously considered quitting [54]. Similarly, McGowan and colleagues found that people were more motivated to be physically active when they became familiar with the protective effect of physical activity on the development of CRC [55]. A promising strategy for delivering key information about CRC risk factors and causation myths could be the integration of educational initiatives into school curricula. This approach, exemplified by the incorporation of cancer education programs into national school curricula, may mitigate delays in cancer diagnosis and improve survival rates. Such interventions have demonstrated efficacy in aiding cancer prevention and early intervention efforts in some Arabic countries like Oman [56, 57].

### Future directions

Targeted educational interventions need to be developed and put into action in order to raise knowledge of CRC and evaluate how such interventions can impact attitudes toward the disease. Promoting awareness through public campaigns can be made more relevant and effective by tailoring interventions based on cultural and religious considerations. For example, campaigns should integrate and discuss the concept of fatalism in the context of Palestinian society. Community involvement in the planning and implementation of interventions can improve their acceptability and effectiveness. In addition, in the era of digital communication, there is a need for investigating the role of digital health interventions, including mobile apps, online platforms, and social media, in fostering CRC awareness and influencing attitudes. Finally, robust research is needed to elucidate the influence of CRC awareness on attitudes toward screening procedures, such as colonoscopy.

### Limitations

This study is subject to certain limitations. The use of convenience sampling may not fully ensure the creation of a representative sample of the Palestinian population, thus constraining the generalizability of the findings. Nevertheless, the large sample size, high response rate, and the diverse regional data collection employed in the study may have mitigated this limitation. Additionally, the decision to exclude individuals from oncology departments and those with medical backgrounds might have potentially led to a reduced number of participants presumed to have high CRC awareness. However, this exclusion was a deliberate choice aimed at maximizing the study’s ability to measure public awareness of CRC. It is also important to note that the study focused on participants’ perceived knowledge and did not assess the awareness of individuals exhibiting actual CRC symptoms. Finally, even though data were collected by trained data collectors utilizing a validated tool, it is challenging to rule out the possibility of having an impact by the subjectivity and self-recall of participants on study interpretations.

## Conclusion

This study identified significant knowledge gaps in CRC awareness among Palestinian adults, specifically concerning CRC signs/symptoms, risk factors, and causation myths. Notably, while individuals who had a high awareness of CRC risk factors and causation myths tended to exhibit a positive attitude toward the disease, the same association was not evident for those with a high awareness of CRC symptoms/signs. Given the suboptimal awareness of all aspects related to CRC, tailored public health campaigns are needed. Such initiatives may have the potential to alter people’s perceptions of CRC, which may help establish a nationwide screening program in Palestine.

## Data Availability

The datasets used and/or analysed during the current study available from the corresponding author on reasonable request.

## Abbreviations

CRC: Colorectal cancer
CAM-MYCS: Cancer Awareness Measure-Mythical Causes Scale
BoCAM: Bowel cancer awareness measure
CI: Confidence interval
OR: Odds ratio
